# The Impact of Electronic Health Record–Based Simulation During Intern Boot Camp: Interventional Study

**DOI:** 10.2196/25828

**Published:** 2021-03-09

**Authors:** Matthew E Miller, Gretchen Scholl, Sky Corby, Vishnu Mohan, Jeffrey A Gold

**Affiliations:** 1 Division of Pulmonary and Critical Care Medicine Oregon Health & Science University Portland, OR United States; 2 Department of Medical Informatics Oregon Health & Science University Portland, OR United States

**Keywords:** electronic health records, medical education, simulation, usability, training

## Abstract

**Background:**

Accurate data retrieval is an essential part of patient care in the intensive care unit (ICU). The electronic health record (EHR) is the primary method for data storage and data review. We previously reported that residents participating in EHR-based simulations have varied and nonstandard approaches to finding data in the ICU, with subsequent errors in recognizing patient safety issues. We hypothesized that a novel EHR simulation-based training exercise would decrease EHR use variability among intervention interns, irrespective of prior EHR experience.

**Objective:**

This study aims to understand the impact of a novel, short, high-fidelity, simulation-based EHR learning activity on the intern data gathering workflow and satisfaction.

**Methods:**

A total of 72 internal medicine interns across the 2018 and 2019 academic years underwent a dedicated EHR training session as part of a week-long boot camp early in their training. We collected data on previous EHR and ICU experience for all subjects. Training consisted of 1 hour of guided review of a high-fidelity, simulated ICU patient chart focusing on best navigation practices for data retrieval. Specifically, the activity focused on using high- and low-yield data visualization screens determined by expert consensus. The intervention group interns then had 20 minutes to review a new simulated patient chart before the group review. EHR screen navigation was captured using screen recording software and compared with data from existing ICU residents performing the same task on the same medical charts (N=62). Learners were surveyed immediately and 6 months after the activity to assess satisfaction and preferred EHR screen use.

**Results:**

Participants found the activity useful and enjoyable immediately and after 6 months. Intervention interns used more individual screens than reference residents (18 vs 20; *P*=.008), but the total number of screens used was the same (35 vs 38; *P*=.30). Significantly more intervention interns used the 10 most common screens (73% vs 45%; *P*=.001). Intervention interns used high-yield screens more often and low-yield screens less often than the reference residents, which are persistent on self-report 6 months later.

**Conclusions:**

A short, high-fidelity, simulation-based learning activity focused on provider-specific data gathering was found to be enjoyable and to modify navigation patterns persistently. This suggests that workflow-specific simulation-based EHR training throughout training is of educational benefit to residents.

## Introduction

The use of electronic health records (EHRs) has expanded significantly over the past 20 years. Spurred by the Health Information Technology Act of 2009 for adoption and meaningful use of the EHR, there was a 6-fold increase in EHR use after over US $19 billion was allocated to facilitate their adoption [1]. Consequently, the EHR is now the central health information storehouse used to facilitate clinical decision making.

With the widespread adoption of EHRs, there have been a number of unintended consequences. The first is the increase in patient harm if the information is not entered, retrieved, or processed correctly, coined e-iatrogenesis [2]. A recent retrospective review showed 2000 medical errors directly related to EHR use over 3 years in the state of Pennsylvania alone, and this may be an underrepresented number given the underreporting of events [3]. Second, there is an increase in provider burnout because of the burden of EHR use [4-6]. The complexity of EHRs has increased the amount of time providers spend documenting outside of work hours, reduced the amount of time spent with the patient, and increased documentation time overall [7-9].

Central to addressing both of these issues is the improvement in EHR education to ensure providers are capable of safe, effective, and efficient use of the EHR in the context of their specific, daily workflow. As a result, multiple groups have developed competencies for EHR training and their integration into medical education; however, effective implementation remains elusive [10,11]. Furthermore, these studies focus primarily on improved efficiency and data entry, although most of the time spent by residents with the EHR focuses on data gathering [12]. Through the use of eye and screen tracking, we have previously demonstrated that there is a lack of a standard approach to use the EHR concerning screen navigation, with only a very small subset of screens used universally by residents. This is associated with a decrease in the number of embedded safety items recognized within simulated EHR charts and subsequent massive variance in perceived diagnosis and plan [13-15]. *Safety items* were defined as data elements that should trigger new diagnoses or clinical management changes if appropriately recognized. Furthermore, these studies identified specific screens and data gathering patterns on screens associated with a greater likelihood of identifying critical patient issues within the charts. These studies not only delineate a framework for metrics to use to design and assess an educational curriculum but also highlight the significance of this variance in patient care.

Multiple challenges with implementing EHR education persist despite the relatively ubiquitous use of the EHR in health care delivery and the growing awareness that EHR use comprises a large portion of a resident's daily work [16]. A number of studies suggest that physicians believe their basic, standard EHR training, typically associated with onboarding when they start their residencies, is inadequate. A recent study suggests that surgery residents spend the first 8 months of their residency becoming proficient with the EHR [17-19]. Residents desire more EHR-related education, which is more likely to be positively received when taught by peers [18,20]. In terms of specific EHR-related education for medical trainees, although there have been some educational interventions to facilitate learning at the medical school level reported in the literature, there is scant literature on educational activities designed to improve resident workflow in the EHR [21]. Residents typically learn EHR skills by emulating their supervisor or peer EHR use, which generally focuses on comprehensive documentation to optimize billing rather than communicating clinical reasoning or quantifying the patient’s clinical status [22].

The utilization of EHR simulations that feature patient records has gained traction as a solution for these problems in EHR education because, as stated by a national consensus conference, simulation is capable of matching EHR training with provider-specific workflow [22-24]. Critical to this is to ensure that the EHR chart has the appropriate degree of realism (which is termed *Fidelity*) to allow for reproduction on workflow. This includes having the appropriate density and quality of data, the ability to house charts in the same system used clinically, to maintain user and system customizations, and to shift charts temporally so that data are current and thus, consistent with the day of activity [24-26]. Our group has previously developed high-fidelity simulated patient cases to assess safe and effective EHR use [13,15,27,28]. Participation in EHR-based simulation improved recognition of embedded patient safety issue recognition upon repeat simulation testing [27]. We have also described the ability to integrate EHR-based simulation into an intern boot camp, demonstrating wide variance in the content of resident-generated notes [29]. Therefore, given the previous data on the lack of standardized use of the EHR and its impact on clinical workflow, we hypothesized that a high-fidelity simulation exercise focused on an ideal EHR navigation strategy would not only be well liked by learners but would also allow for greater standardization of EHR use with a shift toward the use of screens designed to facilitate ideal data gathering.

## Methods

### Cohort and Lesson Plan

Our intervention interns consisted of 71 first-year internal medicine residents at Oregon Health and Science University (OHSU) who completed training and simulation-based learning sessions. There were 33 participants (14 males and 19 females) in 2018 and 38 participants (24 males and 14 females) in 2019. Four participants in 2019 were preliminary neurology residents. All subjects received a dedicated EHR training using high-fidelity simulation-based learning (as described below). The training session occurred during a week-long boot camp in their second or third month of training, the details of which have been previously described [29,30]. Here, we also provide historical data on established workflow from reference residents participating in multidisciplinary simulation for assessing intensive care unit (ICU) safety with regard to EHR use. Reference residents consisted of 33 first-year, 12 second-year, and 13 third-year internal medicine residents. These reference residents used the same or similar simulated charts as the intervention interns. In these studies, residents were assessed for their ability to recognize embedded safety items within the charts; eye and screen tracking were integrated to define navigation patterns and assess the use of specific screens associated with improved identification of said items [14,15]. All participants underwent Epic (Epic Systems Corporation) training as part of their initial onboarding.

Each simulation session performed with our intervention interns consisted of 5-7 participants, 1 instructor, and 1 teaching assistant. Each learner had an individual workstation. The instructor screen was projected to be visible to learners during both guided reviews and debriefing. All subjects completed a survey assessing prior EHR experience and other demographic characteristics at the beginning of the session. The learning activities were divided into 3 sections. In section 1 (duration approximately 1 hour), learners were provided a detailed script on optimal EHR navigation strategies and a number of *high-yield* and *low-yield* screens for effective navigation. These were determined by expert opinion and analysis from previous simulation activities based on the impact of recognizing embedded safety items within simulated charts [15]. The instructor then provided a guided review of a simulated EHR chart demonstrating all aspects of the script and emphasizing the EHR navigation pattern. In section 2, learners were provided a 1-hour didactic on ICU bedside patient presentation skills, though this section was limited to 20 minutes in 2019 because of externally imposed time constraints. In section 3, learners had an independent activity consisting of a 20-minute review of a second simulated ICU patient case. After this, participants in 2018 gave individual mock ICU bedside patient presentations, although this was excluded in 2019 again because of time constraints. A 20-minute group debriefing of the case content concluded the activity, illustrating how the recommended EHR navigation pattern can uncover embedded patient safety issues within the case. The flow of both years’ lessons can be found in Multimedia Appendix 1.

### Simulation Description

Our research group has developed multiple high-fidelity simulated ICU patient charts with accompanying relevant patient data, including vital signs, fluid intake and output, laboratory values, microbiology results, imaging reports, active and inactive medications, active and inactive orders, documentation, and previous encounters. A copy of Epic, which duplicates user preferences without displaying authentic patient charts, is used to host the simulated cases. Cases are copied and transposed forward in time to the date of the simulation, as previously described [13,27]. In addition, screens available in the Epic interface were divided into high- and low-yield categories, as determined by a survey given to senior critical care attending and fellow physicians at the institution and results of previous simulation exercises. Due to copyright conflict, we are not allowed to show these screens or other images of the EHR in this publication.

### Measures

Background demographics, including previous exposure to various EHRs and self-assessment of the facility inpatient EHR navigation ability using a 5-point Likert scale, were collected via a survey immediately before the activity to determine whether any learner-specific factors impacted performance. Individual computer screens were recorded during the solitary review of the second case using open-source software CamStudio [31] to assess the impact of the activity on screen navigation patterns and screens employed. To determine the immediate learner perception of the activity’s utility, global satisfaction and usefulness data for the boot camp were gathered for the 2018 cohort via an anonymous Qualtrics (Qualtrics) survey but given low response rate is excluded. As a result, the intervention interns in 2019 completed an immediate postactivity satisfaction and usefulness survey using a 5-point Likert scale. Finally, to assess the persistence of the perceived benefit of the activity and self-reported EHR use patterns, all intervention interns were assessed again 6 months after the activity via the Qualtrics survey. To eliminate confusion about which screen each question in this survey referred to, we included both screenshots of the specific screens and the screen name.

### Analysis

Screen recordings from the solitary review of the second case were reviewed for the EHR navigation pattern. Excel (Microsoft Corporation) and Prism (GraphPad Software) were used for statistical analyses. Participant use of high-yield screens, low-yield screens, unique screens, and total screens used were compared with historical controls using Pearson chi-square and 2-tailed Student *t* test.

## Results

Intervention group interns included 33 (100%) of the 2018 OHSU first-year residents and 38 (100%) of the 2019 first-year residents. A participant in 2018 was unable to participate in the independent portion of the activity and was therefore excluded from the analysis. A total of 47% (33/71) of the participants were female (Table 1), 67% (48/71) had rotated in the ICU, and 77% (54/77) had experience with the EHR before the activity. When asked to rate themselves on their ability to use the EHR efficiently and comprehensively, intervention interns ranked themselves as *average* with no differences between years.

**Figure 1:**
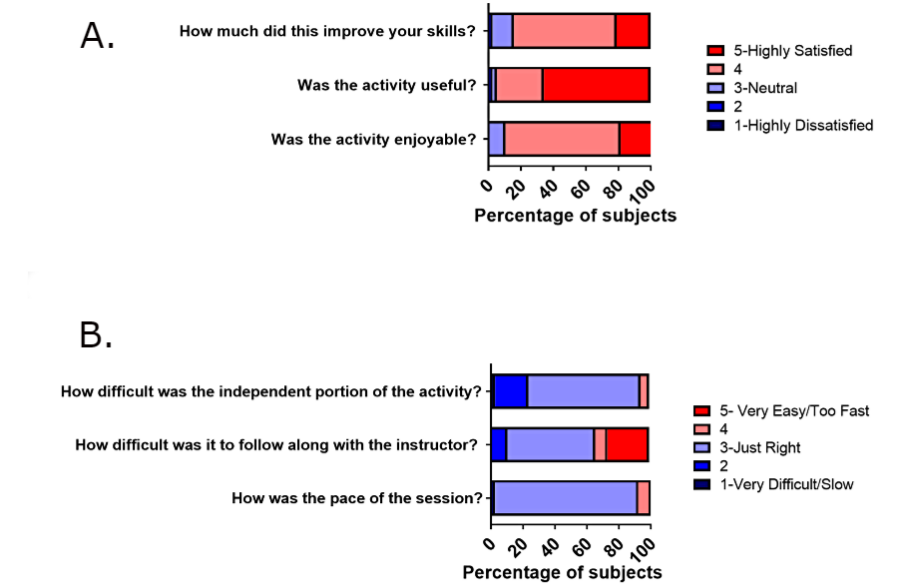
Postactivity satisfaction survey immediately after the lesson. Intervention interns (N=38) were surveyed on a 5-point Likert scale for their impression of the simulation-based learning activity immediately after the session. Panel A: percentage of participants reporting the activity improved their skills, was useful, and enjoyable. Learners found the activity to be helpful and enjoyable. Panel B: percentage of participants reporting the difficulty of the independent portion, following the instructor, and the session’s pacing. Learners found the activity to be appropriately challenging and well-paced.

**Figure 2:**
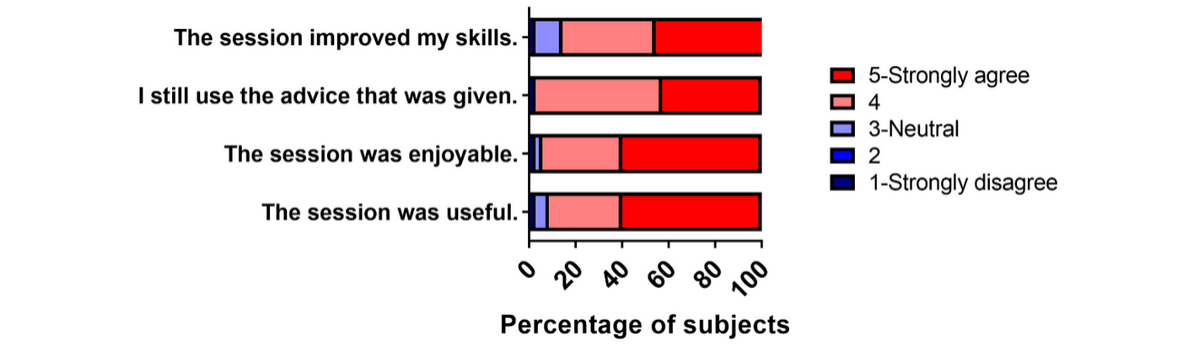
Postactivity satisfaction survey 6 months after the lesson. Intervention interns (n=35, 49%) were surveyed on a 5-point Likert scale for their impression of the simulation-based learning activity 6 months after the session. The graph shows the percentage of participants reporting that the activity was useful and enjoyable, they still use the advice given, and the activity improved their skills. Learners continued to find the activity useful after 6 months of real-world skill use.

A total of 38 (100%) intervention interns in 2019 responded to the satisfaction survey given immediately after the activity. They found the activity to be enjoyable, useful, meaningful, appropriately paced, and appropriately challenging on surveys given immediately after the activity (Figure 1; Multimedia Appendix 2). The qualitative free responses supported the quantitative data (Textbox 1). No correlation was found between any participant characteristics and survey responses (data not shown). A total of 35 (49%) participants responded to the satisfaction survey given 6 months after the activity and found the activity to be useful, enjoyable, and impactful (Figure 2; Multimedia Appendix 2).

**Table 1:** Background data on first-year residents undergoing educational activity.

Question	Value
Female, n (%)	33 (47)
Had previous ICU^a^ experience, n (%)	48 (67)
Had previous experience with our facility’s Epic, n (%)	54 (77)
Had previous experience with Cerner, n (%)	34 (49)
Had previous experience with another facility’s Epic, n (%)	39 (56)
Had previous experience with Allscripts, n (%)	6 (9)
Had previous experience with VistA^b^/CPRS^c^, n (%)	21 (30)
Had previous experience with an EHR^d^ not otherwise listed, n (%)	21 (30)
Self-reported ability to efficiently use any EHR^e^, mean (SD)	3.0 (0.5)
Self-reported ability to comprehensibly use any EHR^e^, mean (SD)	3.0 (0.4)
Self-reported ability to efficiently use facility EHR^e^, mean (SD)	2.8 (0.7)
Self-reported ability to comprehensibly use facility EHR^e^, mean (SD)	2.9 (0.7)

^a^ICU: intensive care unit.

^b^VistA: Veterans Health Information Systems and Technology Architecture.

^c^CPRS: Computerized Patient Record System.

^d^EHR: electronic health record.

^e^Likert scale ranging from 1 (poor) to 5 (excellent).

**Example of free responses to thoughts on the learning activity.**

“Fantastic to help us optimize the EHR...Please have more of these sessions throughout residency”

“Second session going through {patient} on our own, then debriefing was great”

“{It was} very valuable. Wish I’d had a session like this in medical school”

“I{t} was a good time to do {the activity} in the year. {The activity} would not have been helpful during orientation”

“{The second case was a} great case to challenge cognitive biases. The {guided first case} was extremely useful”

“Applicable tidbits & features. Good class involvement”

“some more test cases/examples”

“Practice case was hard, but great learning experience”

“At times couldn’t follow where instructor was clocking-more of a room issue”

We next sought to determine the impact of the program on EHR screen utilization during independent learning activities. Although the average number of total screens viewed by our learners was not significantly different from that of the reference residents (37.8% vs 34.7%; *P*=.17), the average number of unique screens used by our cohort was higher (20.2% vs 17.5%; *P*=.008). As a result, the ratio of total and unique screens tended to be higher in the controls (not shown) and, specifically, the percentage of subjects with a ratio >2, suggesting a high rate of visiting multiple screens multiple times (50% vs 34%; *P*=.06).

**Figure 3:**
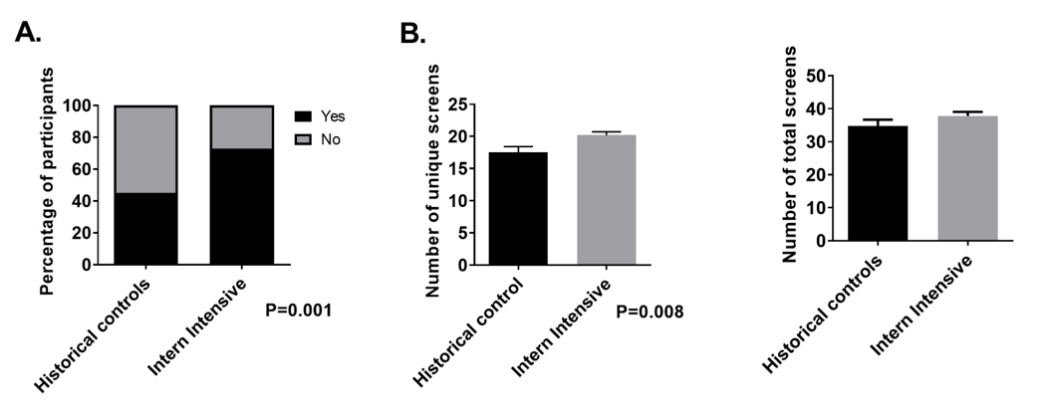
Parameters of screen use. Reference residents (n=62, 100%) and intervention interns with available data (n=70, 99%) had data gathering navigation patterns during postlesson simulation recorded. Panel A: number of reference residents and intervention interns who used the most common 10 screens among all participants. Intervention interns used these most common screens more frequently than participants using previously established workflow (73% vs 45%; *P*=.001). Panel B: number of total screens and unique screens visualized by reference residents and intervention interns. Although there was no difference in total screens used between groups, intervention interns used more unique screens than the reference (20.2 vs 17.5; *P*=.008).

Next, we looked at the 10 most commonly used screens for each cohort. Overall, there was a significant increase in the number of individuals using all 10 of these screens in the intervention group compared with the previously established workflow (73% vs 45%; *P*=.001; Figure 3). Interestingly, this was associated with a slight increase in the number of unique screens viewed (20.2% vs 17.5%; *P*=.008), with no difference in the total screens viewed (37.7% vs 34.7%; *P*=.30; Figure 3).

**Figure 4:**
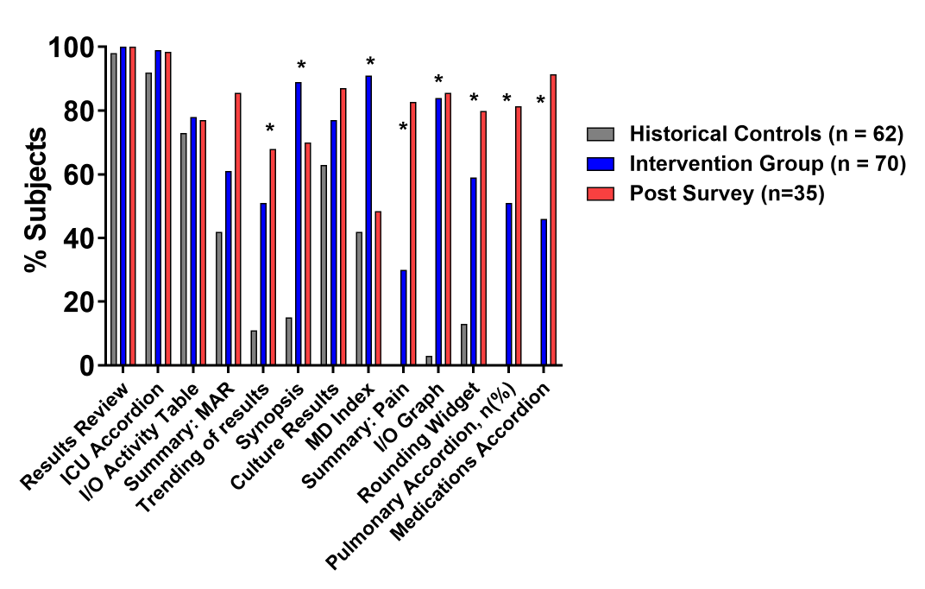
Percentage of reference subjects and intervention interns using high-yield screens and participant self-reported use of high-yield screen 6 months after the intervention. The reference residents (N=62) and intervention interns with available data (n=70, 99%) had data gathering navigation patterns during the independent learning portion of the simulation recorded. Intervention interns used 8 of 13 high-yield screens more frequently by Pearson chi-square as denoted by *P<.05. Intervention interns responded to a survey querying the continued use of high-yield screens 6 months after the lesson (n=35, 49%), with qualitatively maintained uptake. I/O: Intake/output; ICU: intensive care unit; MAR: Medication Administration Record.

**Figure 5:**
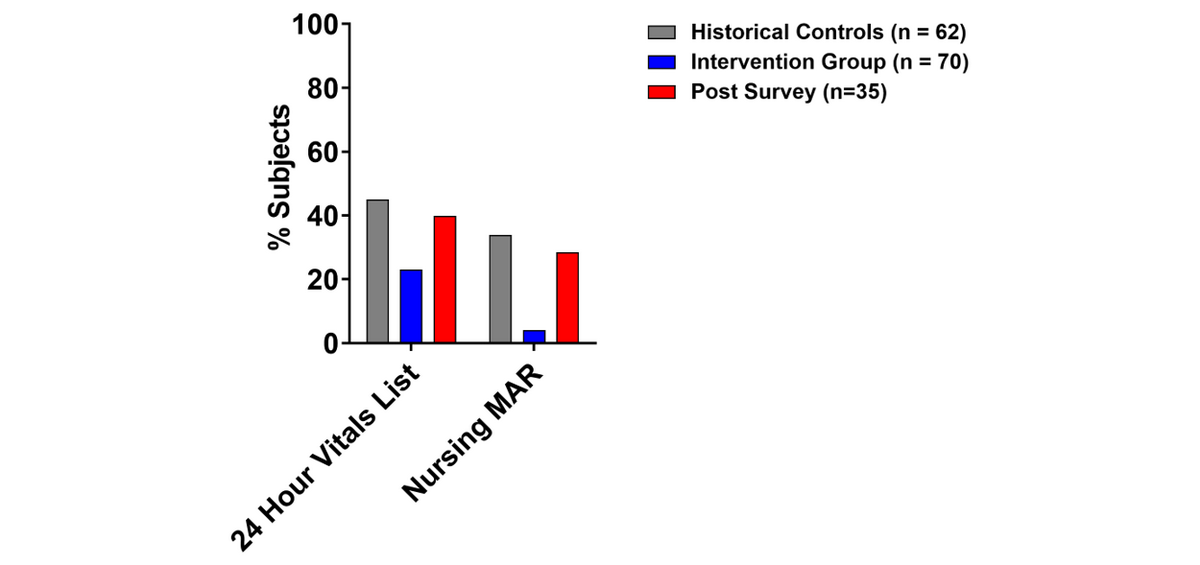
Percentage of reference subjects and intervention interns using low-yield screens and participant self-reported use of low-yield screen 6 months after the intervention. The reference residents (N=62) and intervention interns with available data (n=70, 99%) had data gathering navigation patterns during the independent learning portion of the simulation recorded. Intervention participants used low-yield screens less frequently than historical controls by Pearson chi-square (*P*<.05). Intervention interns responded to a survey querying continued use of high-yield screens 6 months after the lesson (n=35, 50%); decreased use of low-yield screens was not sustained. I/O: Intake/output; ICU: intensive care unit; MAR: Medication Administration Record.

Of the 11 high-yield screens recommended during the guided review, 8 were used statistically significantly more by our intervention interns (Figure 4 and Multimedia Appendix 2). When we assessed the self-reported use of these screens at 6 months, we observed continued high use of these screens. Conversely, when we looked at the ability of the activity to discourage the use of 2 low-yield screens, we observed the use of 2 low-yield screens to be significantly lower in the intervention interns than in the reference. However, discouragement of low-yield screens attenuated over time, with increased self-reported use 6 months after the activity (Figure 5). Finally, when we looked at predictors of high-yield screen use during the simulation, only prior ICU experience predicted the use of graphing functions to review laboratory data (42.8% vs 18.8%; *P*=.03). Otherwise, none of the other variables (sex and prior EHR use and experience) predicted screen use (data not shown).

## Discussion

In this study, we report the development of a novel, dedicated 2-hour EHR training focused on physician workflow while preparing to evaluate a patient at the beginning of the day (prerounding) using high-fidelity simulation-based learning, with special attention to high-yield and low-yield screens available in the EHR interface. We observed high and sustained learner satisfaction with the activity, which was associated with significant and sustained changes in navigation patterns with respect to the established workflow previously seen in reference residents. Most importantly, these perceptions were sustained 6 months after the activity.

In contrast to previous studies where providers have historically reported low engagement and enjoyment with traditional EHR-based education, our study participants reported high and persistent learner satisfaction; they also perceived usefulness upon immediate postactivity assessment, likely secondary to the use of high-fidelity simulations as the model of instruction [32]. In addition, most EHR education traditionally focuses on the basic functionality of the clinical information system, whereas our lesson focused on practical, systematic approaches to data gathering consistent with learners’ realistic workflow. Qualitative comments elicited from participants indicated that the experience was enjoyable and pertinent because of factors such as challenging and realistic cases, layout of the lesson (guided review of a case, solitary review of a case, and then group debrief), learner engagement during the guided review, focus on systematic data extraction, and timing of the lesson a few months after real-world exposure.

Although a number of studies document the impact of EHR-based onboarding on provider satisfaction, few have documented its impact on the way they actually proceed to use the EHR, specifically their EHR screen navigation habits. Simulation has been used for basic EHR education, and a recent study documented the impact of simulation training on the use of a specific data visualization screen and a single information retrieval tool [23,33]. Our study expands on these findings by focusing on changes in the entirety of participant EHR screen navigation patterns after high-fidelity simulation-based learning. Overall, our intervention was associated with an increase in the standardization of EHR use, as evidenced by a near doubling in the number of individuals using the most common screens. Furthermore, the increase in the total number of unique screens employed, with little change in total screens, supports a shift toward data gathering along a scripted progression of different screens within the EHR rather than alternating between a few screens repeatedly. This has potential impacts on information retrieval precision and cognitive processing, as returning to a previously viewed screen within an EHR has been associated with cognitive overload [34,35].

Standardization in screen use was associated with the increased use of high-yield screens and decreased use of low-yield screens during the independent learning portion of the activity. Perhaps more importantly, intervention interns retained these skills 6 months after the session. These results are consistent with a previous study, which demonstrated increased use of a specific EHR-based tool after a simulation-based exercise as assessed through user logs [23]. Unfortunately, EHR user logs were unsuitable for our analysis, as the information collected by audits at our institution does not include the users’ contextual activity. Our learning focused on navigation patterns while data gathering prerounding, but user audit logs would be unable to distinguish this activity from that of data entry or documentation. Audit logs also lack information on timing with respect to patient interaction. Although our lesson focused on prerounding on new patients, logs would also capture all of the EHR navigation conducted during the day, including prerounding on known patients, assessing new patients, data gathering to address a change in clinical status, and review during preparation to transfer care (sign-out). However, our follow-up survey suggests that most of the participants continued to find high-yield screens valuable. Thus, overall, the data collected in this study suggests not only that our activity was able to modify participant behavior effectively but also that these changes were sustained long beyond the activity.

Next, we sought to determine whether any participant characteristics impacted either user satisfaction or adoption of EHR best practices. Overall, prior EHR use, sex, and perceived comfort level with EHRs generally and our EHR specifically had no impact on learner satisfaction or performance. However, learners who had already rotated in the ICU showed increased use of the graphing functions of the EHR to visualize laboratory data. This association suggests that although this type of activity is relatively generalizable, some specific EHR skills are still better adopted when placed in the context of actual experience. This is consistent with feedback from learners in the free-text comments of the survey. However, it must be stressed that these studies were specifically conducted after all learners had completed 2 months of internship and thus already had significant experience with the intern workflow in general. It remains to be determined whether this activity would have the same impact if implemented at the very beginning of residency, integrated into their initial EHR onboarding activity.

This study has some important limitations. The first is the use of an established workflow from reference residents for comparators of screen navigation rather than a randomized control. However, as our reference residents participated in simulations using the same simulated charts; were assessed during their ICU rotations; and comprised trainees of all levels with, therefore, greater clinical and EHR exposure, they represent a more expert group of users compared with the intervention interns. Despite a more expert established baseline, we were still able to detect the effect of training. The second is a lack of preactivity assessments. Assessment of navigation patterns before and after the educational activity would have provided stronger support for causality in between-screen navigation pattern change in the intervention. Unfortunately, because of external time constraints, we were unable to perform a preactivity navigation pattern assessment. Similarly, we were limited to self-reporting via a web-based survey to assess retention, as the interns did not have the time to participate in additional simulations, and there was no reliable way to query the EHR to assess real-world screen navigation. Finally, this exercise focused purely on information retrieval. Although this is the most common activity performed by interns in the EHR, there are other important domains of EHR use, including optimization of data entry (eg, note writing) and managing in-basket alerts, that were not addressed [12].

In conclusion, our study presents a novel, short, high-fidelity EHR-based simulation, with special attention to provider-specific workflow during prerounding as opposed to EHR functionality, as an agreeable and effective educational activity. Learners found the activity enjoyable and useful both immediately and on reassessment 6 months after the activity. We found navigation patterns to closely match expert recommendations after the activity. These findings are important given the historical inadequacy of EHR training. The ability to deliver this content in a short time frame allows for the rapid expansion of this methodology not only during onboarding but also throughout the continuum of their training. Future directions may focus on using this technique to optimize other resident interactions with the EHR.

## Appendix

Multimedia Appendix 1 Lesson plan.

Multimedia Appendix 2 Supplementary data tables.

## Abbreviations

EHR: electronic health record
ICU: intensive care unit
OHSU: Oregon Health and Science University
