# Supplementary figures and images for: The Impact of Electronic Health Record–Based Simulation During Intern Boot Camp: Interventional Study
Source: JMIR Med Educ. 2021 Mar 9;7(1):e25828. doi: 10.2196/25828 (PMC8081274; doi:10.2196/25828)

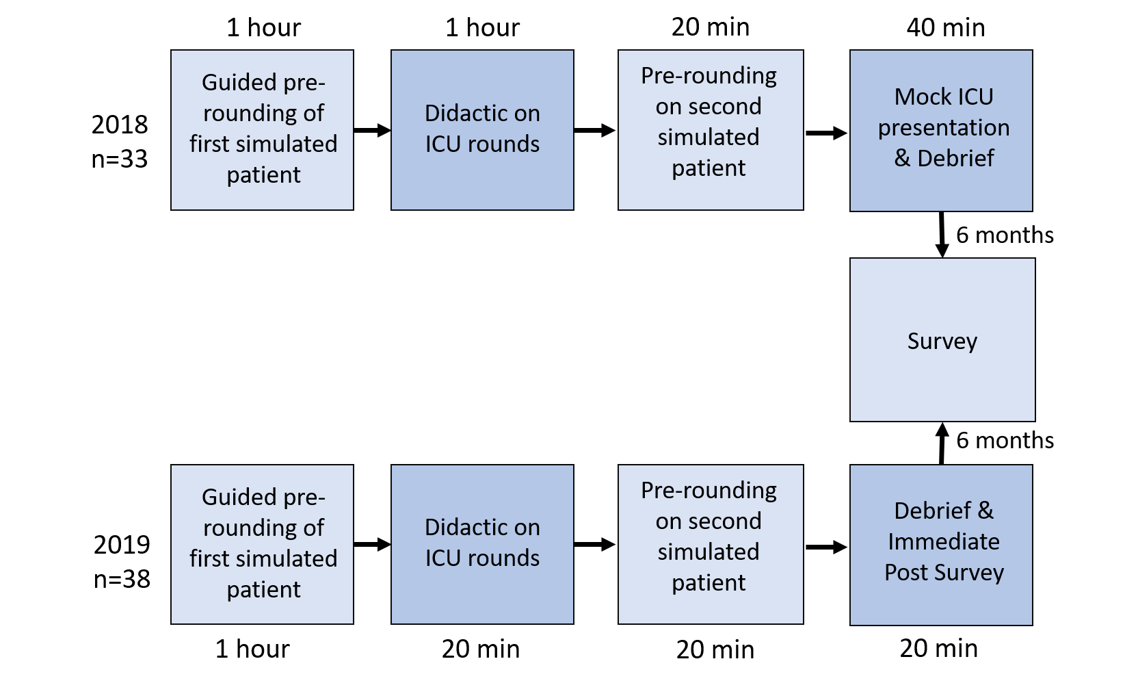

Supplement: Multimedia Appendix 1 [file mededu_v7i1e25828_app1.png]
