# Supplementary material for: The Impact of Electronic Health Record–Based Simulation During Intern Boot Camp: Interventional Study
Source: JMIR Med Educ. 2021 Mar 9;7(1):e25828. doi: 10.2196/25828 (PMC8081274; doi:10.2196/25828)
Supplement: Multimedia Appendix 2 [file mededu_v7i1e25828_app2.docx]

**Supplementary Table 1-Results of Immediate Post Session Survey.** Data collected with a 5-ppint Likert scale and presented as Mean (std). ^1^Likert scale from 1 (Not at all) to 5 (Extremely), ^2^Likert scale from 1 (Very slow) to 5 (Very fast), ^3^Likert scale from 1 (Very difficult) to 5 (Very easy). N=38

| How enjoyable was this session? ^1^ | 4.1 (0.5) |
| --- | --- |
| How useful was this session? ^1^ | 4.6 (0.7) |
| How much did this session improve your skills? ^1^ | 4.1 (0.7) |
| How was the pace of this session? ^2^ | 3.1 (0.3) |
| How difficult was it to follow along with the instructor? ^3^ | 3.6 (0.9) |
| How difficult was the independent portion? ^3^ | 2.8 (0.5) |

| I found the session useful | 4.5 (0.9) |
| --- | --- |
| I found the session enjoyable | 4.5 (0.8) |
| I still use the advice I was given | 4.3 (0.8) |
| The session impacted by ability to effectively gather information | 4.3 (0.9) |

**Supplementary Table 2 3. Acceptability Survey at Six Months after Activity.** Data collected using a 5-point Likert Scale from 1 (Strongly Disagree) to 5 (Strongly Agree). Data presented as mean (Std). N=35

|  | **Historical Controls (n = 62)** | **Intervention Group (n = 70)** | **p-Value** |
| --- | --- | --- | --- |
| **High Yield Screen, N (%)** |  |  |  |
| ICU Accordion | 57 (92) | 69 (99) | 0.068 |
| Summary: MAR | 28 (42) | 43 (61) | 0.061 |
| Trending of results | 7 (11) | 36 (51) | <0.001 |
| Synopsis | 9 (15) | 62 (89) | <0.001 |
| Culture Results | 39 (63) | 54 (77) | 0.074 |
| MD Index | 26 (42) | 64 (91) | <0.001 |
| Summary: Pain | 0 (0) | 21 (30) | <0.001 |
| I/O Graph | 2 (3) | 59 (84) | <0.001 |
| Rounding Widget | 8 (13) | 41 (59) | <0.001 |
| Pulmonary Accordion | 0 (0) | 36 (51) | <0.001 |
| Medications Accordion | 0 (0) | 32 (46) | <0.001 |
| **Low Yield Screen** |  |  |  |
| 24 Hour Vitals List | 28 (45) | 16 (23) | 0.007 |
| Nursing MAR | 21 (34) | 3 (4) | <0.001 |

**Supplementary Table 3-Use of High Yield and Low Yield EHR Screen During Simulated ICU Pre-Rounds**. Intervention Group and Historical Controls compared with Chi-Square Independence Test
